# Decrease in Nitric Oxide Production as a Key Mediator in the Pathogenesis of Preeclampsia and a Potential Therapeutic Target: A Case-Control Study

**DOI:** 10.3390/biomedicines10102653

**Published:** 2022-10-20

**Authors:** Maciej W. Socha, Martyna Stankiewicz, Krzysztof Żołnieżewicz, Oskar Puk, Mateusz Wartęga

**Affiliations:** 1 Department of Perinatology, Gynecology and Gynecologic Oncology, Faculty of Health Sciences, Collegium Medicum in Bydgoszcz, Nicolaus Copernicus University, Łukasiewicza 1, 85-821 Bydgoszcz, Poland; 2 Department of Obstetrics and Gynecology, St. Adalbert’s Hospital in Gdańsk, Copernicus Healthcare Entity, Jana Pawła II 50, 80-462 Gdańsk, Poland; 3 Department of Pathophysiology, Faculty of Pharmacy, Collegium Medicum in Bydgoszcz, Nicolaus Copernicus University, M. Curie-Skłodowskiej 9, 85-094 Bydgoszcz, Poland

**Keywords:** preeclampsia, hypertensive pregnancy, gestational hypertension, nitric oxide, NO, pregnancy complications

## Abstract

Pregnancy-induced hypertension (GH) complicates 6–10% of all pregnancies and, in 2019, was responsible for approximately 28,000 deaths. The most common cause of gestational hypertension is pre-eclampsia (PE), which afflicts 2–8% of all pregnancies and is one of the three leading causes of maternal morbidity and mortality worldwide. The aim of this study was to clarify how NO metabolism changes during the course of PE. Due to the short half-life of NO, we measured the concentrations of its stable metabolites, nitrite and nitrate (NOx). Out of 100 enrolled patients: 58 pregnant women with a diagnosed early form of PE formed a study group, and 42 healthy pregnant women formed a control group. NOx concentrations were significantly lower in the PE group than in the control group, with mean values of 5.33 and 27.64 μmol/L, respectively (*p* < 0.0001). The decrease in NO is most likely the result and mediator of systemic endothelial dysfunction. The impairment of NO metabolism in PE appears to play an important role in its pathogenesis. Therefore, it is a potential therapeutic target.

## 1. Introduction

In 2019, maternal disorders among women aged 10–54 years afflicted approximately 112 million women worldwide and were the cause of 196,000 deaths [1,2]. In these numbers, gestational hypertension (GH), which complicates approximately 6–10% of all pregnancies, was responsible for 14% of deaths [1,2]. Notably, GH is associated not only with significant maternal mortality but also significant fetal mortality [1]. GH is defined as systolic blood pressure >140 mmHg and/or diastolic blood pressure >90 mmHg, and it is related to an increased risk of cerebrovascular events, disseminated intravascular coagulation (DIC), organ failure, and placental abruption [2,3]. Hypertensive disorders in pregnancy most often occur in the form of pre-eclampsia (PE), which afflicts 2–8% of pregnancies and is one of the three leading causes of maternal morbidity and mortality worldwide [2,3]. PE is defined as de novo hypertension present after 20 weeks of gestation combined with proteinuria (>300 mg/day) and/or other maternal organ dysfunction, hematological complications, uteroplacental dysfunction, or fetal growth restriction [3]. Pre-eclampsia can be complicated by DIC, HELLP syndrome, liver hemorrhage, placental abruption, pulmonary edema, and stroke [3,4,5].

PE is a major problem in obstetrics because little is known about its pathogenesis, and there is no preventative treatment. Nevertheless, in the last few decades, many theories explaining the etiopathogenesis of pre-eclampsia have been developed, such as uteroplacental ischemia, uterorenal reflex, immunological, prostaglandin, osmotic and oncotic theory, peptide renin–angiotensin–aldosterone system (RAAS), vestibular peptide, and natriuretic and stress theories [6].

The aforementioned theories most likely describe only a part of the complex PE pathogenesis, which is an effect of systematic inflammation. The source of this general inflammatory response may be different but, most often, it begins as regional inflammation in the placenta due to impaired placentation [7,8,9].

For decades, the leading model of PE development was the two-stage placental model; however, knowledge regarding PE pathogenesis advanced through the years as researchers reported many environmental factors not associated with the placenta, which can initiate a systemic inflammatory response [8,9]. These factors, such as monosodium urate, cholesterol, and high concentrations of sodium or glucose, through activation of nuclear factor κB and NOD-like receptor family and pyrin domain-containing protein 3 (NLRP3) inflammasome can lead to the development of PE, sometimes without any observable pathologies in the placenta [7,8]. Recently, the multistage model of PE development was created, indicating that poor placentation leading to systemic inflammation and endothelial dysfunction is the most common pathological pathway leading to PE development [9].

In order to understand this stage of PE development, it is necessary to evaluate the molecules responsible for the contraction and relaxation of blood vessels; nitric oxide (NO) is the main vasodilator in the human body. Therefore, for decades, research has been carried out to determine its concentrations and the role in PE pathogenesis [10,11,12,13,14]. Nevertheless, the results of these experiments have been inconclusive. Some of them showed increased NO concentrations in women with PE, some showed no changes compared with healthy pregnant women, and some showed a significant decrease in NO concentrations [10,11,12,13,14]. Furthermore, the number of participants was relatively low and methodology differed, especially in the case of approach to lowering the impact of dietary nitrates. These studies included only approximately 10–30 patients with PE. Therefore, there is a great need for studies with standardized methodology and larger study samples.

With this in mind, we conducted a clinical study to determine how the production of NO changes in patients with the early onset PE in comparison to healthy pregnant women. We aimed to clearly define the direction of changes in NO metabolism in PE and to facilitate the precise determination of the role of NO in the pathogenesis of PE.

## 2. Materials and Methods

### 2.1. Study Design

We measured NO metabolite concentrations in blood samples obtained from patients with PE diagnosed in gestational age in the range of 28 + 0 to 33 + 6 weeks, which met the criteria of the early onset PE, that is, development of PE before the 34th week of gestation. Results obtained from that group were compared with those from matched controls, who were healthy pregnant women of similar gestational age. All participants were patients of the Department of Obstetrics, Women’s Diseases and Gynecological Oncology of Ludwik Rydygier Collegium Medicum in Bydgoszcz of the Nicolaus Copernicus University in Toruń, placed in Jan Biziel University Hospital no. 2 in Bydgoszcz. Blood samples were collected between September 2014 and September 2015. The analysis of samples was performed within three months after collection.

### 2.2. Subjects

The study included 100 women hospitalized in the Department of Obstetrics, Women’s Diseases and Gynecological Oncology of Ludwik Rydygier Collegium Medicum in Bydgoszcz of the Nicolaus Copernicus University in Toruń. The study group consisted of 58 pregnant women with diagnosed PE, and the control group consisted of 42 healthy pregnant women admitted to the clinic due to an upcoming term of physiological childbirth. The study size is a result of the number of patients with early onset PE admitted to the department within a year and inclusion/exclusion criteria.

Patients were classified according to the criteria proposed by the National High Blood Pressure Education Program Working Group on High Blood Pressure in Pregnancy, that is, systolic blood pressure greater than 140 mmHg or diastolic blood pressure greater than 90 mmHg in a woman normotensive before the 20th gestational week, accompanied by proteinuria greater than 300 mg of protein in a 24 h urine sample [15]. Each patient also had Doppler flow tests in the umbilical vessels and the fetal brain’s middle artery, and the concentration of uric acid in the serum was assessed. In addition, all patients had urinalysis performed to verify proteinuria. However, 24 h urinary protein collection assessment was not analyzed in all women due to labor. Therefore, these patients were not diagnosed with proteinuria higher than 300 mg/day, although fast tests revealed proteins in their urine samples, and these patients were diagnosed with PE.

Matching and inclusion/exclusion criteria were: single pregnancy; fasting before sample collection; no diagnosis of preterm labor, cervical insufficiency, placenta previa, or other obstetric diseases; no chronic diseases that may have influenced the results of this study, e.g., cardiovascular or nephrological diseases.

Each patient had a blood sample collected only once, and there were no previous measurements or follow-up.

### 2.3. Sample Collection and Measurement

Due to the fact that the half-life (T1/2) of nitric oxide is 1 to 30 s, after which it is oxidized to nitrite (NO^2−^) and then nitrate (NO^3−^), its concentration in the blood can be analyzed only by an indirect method involving the assessment of the number of its metabolites. In this study, previously frozen EDTA plasma was analyzed using a colorimetric test. A Total Nitric Oxide Assay Nitrite/Nitrate (NO^2−^/NO^3−^) kit (Cat. No. KGE 001) from R&D Systems (Minneapolis, MN, USA) was used.

When making nitrate determinations, we considered dietary exposure to nitrate. Dietary nitrates have been found to increase base plasma nitrate concentration at least two- to three-fold in case of intake of dietary products with high nitrate concentrations [16]. The half-life of dietary NO^3−^ is approximately 5 h. Therefore, in this study, plasma samples were collected from patients approximately 10 h after the last meal to exclude the effect of dietary nitrate [16]. We had scarce information about patient diet, but approximately 24 h before sample collection, patients ate only standard hospital meals and were instructed not to have additional snacks or meals. Blood samples were collected a couple of days before labor.

All stages of the test were performed in accordance with the instructions provided with the R&D Systems kit.

### 2.4. Risk of Bias Assessment

The risk of bias was assessed with the use of the CLARITY Group tool to assess the risk of bias in case-control studies. According to the tool, there was generally a low risk of bias, with a higher risk of bias regarding exposure (we could not be certain that patients were honest about their diet and lifestyle) and matching (it was impossible to identify all prognostic variables in the case of PE as its pathogenesis is not fully comprehended).

### 2.5. Statistical Analysis

The statistical analyses were performed using the PQStat statistical package, version 1.6.6.118.

We used standard statistic methods, such as Student’ *t*-test, chi^2^ relationship test, logistic regression model, etc.

The test probability at the level of *p <* 0.05 was assumed as significant, and the test probability at the level of *p <* 0.01 was assumed as highly significant.

## 3. Results

All patients in the study group met the PE diagnostic criteria. However, only 53.5% of the women had been diagnosed with proteinuria. Other patients met the PE criteria due to other maternal organ dysfunction, hematological complications, uteroplacental dysfunction, or fetal growth restriction. A vital factor lowering the percentage of patients with proteinuria higher than 300 mg/day was a disruption of the 24 h urine collection by labor in some cases. None of the patients from the control group had hypertension or proteinuria.

The patient characteristics are shown in Table 1. In the group of patients with gestational hypertension, the mean age was 29.71 years (median: 29), and the results ranged from 18 to 42 years. In the group without diagnosed hypertension, the mean age was 27.74 (median: 28), and the range of results was from 19 to 38 years. Neither group significantly differed (*p >* 0.05) in terms of age. Thus, despite recognizing age as one of the risk factors for the development of gestational hypertension, in this study, no correlation was found between age and PE occurrence (*p >* 0.05).

As shown in Table 1, there were no statistically significant differences in patient fertility or previous miscarriages.

The data are presented in the format M + SD, where M is the mean value, and SD is the standard deviation. Some parameters, e.g., miscarriages, are given as the number of patients within the group.

Interestingly, although there is a common belief that obesity is a significant risk factor for hypertension, in this study, there was no correlation between high BMI and PE incidence (*p >* 0.05).

The incidence of comorbidities significantly differed between the studied groups (*p <* 0.01). Concomitant diseases occurred in 36.2% of cases in the PE group and 11.9% of cases in the control group. The logistic regression model performed during the statistical analysis proved that the incidence of comorbidities in pregnant women increased the risk of PE by over 4.5 times. The presence of other diseases could be considered a significant determinant of the appearance of the early onset PE (*p <* 0.01). The other considered factors did not show statistical significance in the prediction of disease occurrence.

In the PE group, the average body weight of a newborn was 2652 g (median: 2742.5), and the range of results was from 790 to 4440 g. In the control group, the average weight of newborns was 3117.38 g (median: 3390), and the range of results was from 890 to 4350 g. Thus, newborn weight was significantly lower in the PE group than in the controls (*p* < 0.01). We estimated that approximately 30% of PE women had small for gestational age (SGA) fetuses or fetal growth restriction type 1 (FGR1).

There was no statistically significant difference in newborn sex between the groups.

**Figure 1:**
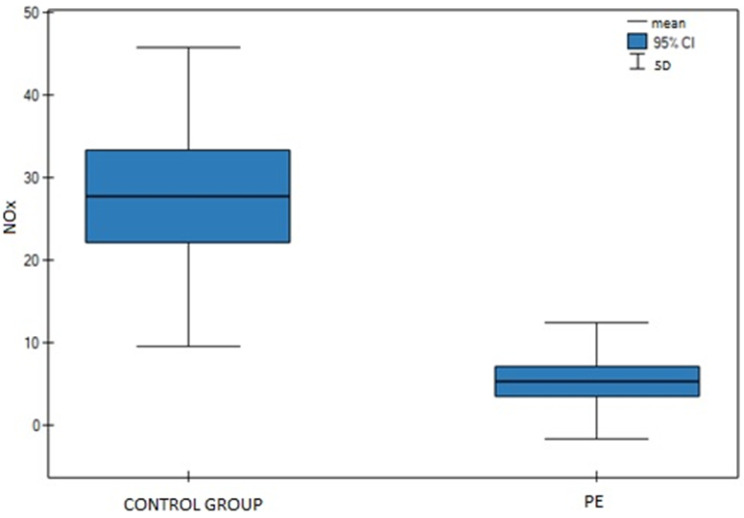
Mean NOx concentrations.

In the PE group, the mean nitrite and nitrate (NOx) concentration, as shown in Figure 1, was 5.33 µmol/L (median: 2.55 µmol/L), and the results ranged from 0 to 33.87 µmol/L. In the control group, the mean NOx was 27.64 µmol/L (median: 22.84 µmol/L), and the results ranged from 5.76 to 100.42 µmol/L (Figure 1). The difference between groups was statistically significant (*p <* 0.01). The measurement results are summarized in Table 2.

## 4. Discussion

Considering the dramatic course and numerous complications of PE, which are dangerous for both mothers and fetuses, over the past decades, many studies have focused on the early detection of predisposition to the development of this disease. However, most of the tests proposed for early PE detection are either extremely difficult and expensive to perform or not confirmed by further scientific research, as we observed. Perhaps, to find a simple PE marker allowing for the early determination of women with a high probability of developing PE, one should refer to the etiopathogenesis of gestational hypertension.

Although both advanced age and high BMI are considered PE risk factors, the logistic regression model revealed no increase in PE risk for age or BMI in this study. Only comorbidities appeared to be an important risk factor of PE development. Based on our and other clinicians’ experiences, this shows that this disease often occurs among patients with low PE risk when considering the standard risk factors. However, it may have been the result of the small size of the study group.

Our previous studies indicated that the vital element in PE pathogenesis is impaired spiral arteries remodeling and, therefore, pathological placentation [7,8]. It has been reported that NO plays an essential role in placentation and, as the main vasodilator in the human body closely associated with vascular endothelium, it plays a crucial role in the adaptation of women’s cardiovascular system during pregnancy when NO concentrations significantly increase [10,16,17,18,19,20,21]. During PE, pathological remodeling occurs, which leads to impaired blood flow, local hypoxia, and inflammation [7,8]. Considering that the NO produced in endothelial cells interacts with vascular smooth muscle cells, we hypothesized that endothelial NO plays a crucial role in the pathological remodeling of spiral arteries and impairment of arteries’ tone regulation, processes leading to the development of PE. In favor of that hypothesis, animal experiments have shown that infusion of an NOS inhibitor causes symptoms similar to those observed in PE [16,22]. Moreover, studies on pregnant rats have shown the development of hypertension and fetal growth restriction (FGR) after the administration of L-nitro-arginine methyl ester hydrochloride, a compound that effectively inhibits neuronal, endothelial, and induced NOS [17,22]. FGR is defined as fetus weight below the 10th percentile. Seligman et al. proved that the NO2 level in the serum of patients diagnosed with pre-eclampsia is inversely proportional to the level of systolic and diastolic blood pressure [16,23]. However, as it was described before, not all findings are consistent, and some studies have shown no change or even an increase in NO concentrations in the course of PE [10,11,12,13,14,24]. However, this may have been the result of a methodological error, as the authors of some studies stated that they had no impact on patient diet, and some did not consider dietary nitrates at all. In favor of such a conclusion, only in studies in which blood samples were taken after fasting or introduction of a low nitrate diet were the NOx levels of patients with PE lower than in healthy pregnant women or nonpregnant women. Dietary nitrates were found to increase the base plasma nitrate concentration at least two- to three-fold in the case of intake of dietary products with high nitrate concentrations [16]. That is why consideration of dietary nitrate exposure when designing an experiment is very important.

In our study, NOx concentrations were significantly lower in the PE group than in the controls, which proves that NO production decreases during the course of PE. This indicates that NOx measurement may be able to distinguish patients with PE at the early stage of pregnancy; therefore, it can be a useful screening test for PE. However, in our study, blood samples for NOx analysis were taken during the gestational age, as indicated in the table, prior to labor, not earlier; therefore, further investigation is needed in order to confirm NOx changes at earlier stages of pregnancy.

The early designation of PE or a high-PE-risk group is very desirable and clinically meaningful, as it can allow for early introduction of proper treatment.

Acetylsalicylic acid (ASA), despite no vasodilatory effect, is currently used in the prevention of PE. It acts by irreversible inhibition of the constitutive cyclooxyrgenase (COX-1) of platelets and weakens thrombinogenesis. Thus, it gives an antiaggregation effect. At higher doses, it acts as an antithrombotic agent through antagonism of vitamin K. A multicenter, international study conducted by the world’s leading perinatal medicine centers, including Nicolaides, described a significant reduction in PE incidence in women at high risk after ASA treatment compared with the placebo group [25]. However, ASA side effects, such as damage to the gastric mucosa with bleeding, thrombocytopenia, peptic ulcer formation, and hypoproteinemia, should not be forgotten. It should also be remembered that ASA inhibits the effect of some antihypertensive drugs, e.g., beta-blockers, and that in the third trimester, the drug should be discontinued to prevent premature closure of the ductus arteriosus [26,27].

The latest reports in the literature show the effectiveness in reducing PE occurrence among pregnant women with diabetes, obesity, or polycystic ovary syndrome by introducing metformin between weeks 5 and 18 of pregnancy and continuing therapy until the labor, probably due to increased perfusion of the placenta [28]. Treatment with metformin was associated with a higher risk of gastrointestinal side effects, such as diarrhea, nausea, and emesis [28].

Considering the aforementioned data, changes in NOx levels indicate problems with vasoconstriction, and patients with impaired regulation of blood flow are at higher risk of PE. Additionally, if it is proven that there are NOx changes at the very early stages of PE, its measurement may be able to distinguish women with PE before we can clinically diagnose the disease. This is the high-risk group; detection of patients from this group will allow for the introduction of proper treatment before any symptom occurrence. Our findings indicate that the decrease in NO production is strongly associated with PE, and it is most likely an important element of its pathogenesis. Therefore, NO supplementation by its donor can be an interesting approach in preventing and treating PE, especially when considering that the placenta grows with the fetus, and active remodeling and creation of new blood vessels occur until the last weeks of pregnancy. The aim of NO donor treatment is the facilitation of physiological development of the placenta and proper remodeling and angiogenesis in its newly created parts, which will allow the fetus to be properly nourished and reduce the production of proinflammatory chemokines. This limits inflammation in the placenta and most likely reduces the systemic inflammatory response. Importantly, this type of treatment can reduce the incidence of PE complications, e.g., HELLP syndrome, which is the most common cause of maternal and fetal mortality during the course of PE. Furthermore, improved blood circulation in the placenta supports fetal development and reduce neonatal complications related to preterm labor, which is often a result of PE or eclampsia.

Although NO donors appear to be a promising treatment strategy, their introduction may be difficult due to dangerous side effects. The NO donors most commonly used in medicine are nitroglycerine and isosorbide dinitrate. However, both drugs are pregnancy category C. Importantly, clinicians have reported cases when NO donors helped to maintain healthy pregnancy, preventing PE without harming the fetuses [29,30,31]. Interestingly, in the study by Razik et al., isosorbide mononitrate was administered via the vagina in the form of 20 mg tablets [30]. This may indicate that intravaginally administered NO donors improve uteroplacental circulation, stopping following inflammation and PE development. Other NO donors, such as nitrosothiols, most likely are not a good therapeutic option, as it was reported that the decomposition of nitrosothiols, and thus the release of NO, is impaired in PE [32]. Recently, a new group of NO donors was developed that is called NO hybrids. Drugs from this broad group, such as NO-NSAID, may have unique properties, which might allow them to be safer for a certain group of patients, e.g., pregnant women. However, they need further investigation to confirm their effectiveness and safety.

The beneficial role of NO donors in PE treatment was proved in a recent work by Gupta et al., who demonstrated an improvement in Doppler velocimetry in the umbilical artery and the uterine artery after using nitroglycerin in pregnant patients diagnosed with GH and FGR [33]. Similar results were obtained in the study conducted by Nakatsuka et al., in which an early diastolic notch was observed in the uterine artery [33,34]. However, these studies have not reported hemodynamic changes in the fetal middle cerebral artery.

In our study, the average body weight of newborns of women with PE was significantly lower than the average body weight of newborns of healthy women. This indicates a placenta dysfunction in the course of PE, a condition which, considering the data mentioned above, can be mitigated by treatment with NO donors.

The limitations of this study are the small sample of participants, lack of comparison with healthy nonpregnant women, lack of correlation of blood pressure with NOx concentration, and the fact that NOx concentrations were measured only once, not systematically during the pregnancy. Nevertheless, this study shows that NO concentrations significantly decrease in the course of PE and that NOx measurement can be a useful screening test. Additionally, there was a higher risk of bias in terms of exposure, as we could not be certain that patients were honest about their diet and lifestyle, and in the case of matching, as it was impossible to identify all prognostic variables in the case of PE as its pathogenesis is not fully comprehended. In other points of the study, the risk of bias was low.

## 5. Conclusions

Typical PE risk factors such as age, BMI, and being in the first pregnancy were not associated with increased PE incidence. Therefore, they did not differentiate PE patients from healthy pregnant women. This advocates for the need for a precise screening test, as it is hard to predict PE occurrence. Comorbidities increase the risk of PE by over 4.5 fold. NOx concentrations significantly decrease in the PE course, which indicates that decreased production of NO is an element of PE pathogenesis. The decrease in NO is most likely the result and mediator of the systemic endothelial dysfunction. NOx measurement allows clinicians to distinguish patients with early onset PE. Therefore, it can be a diagnostic test and possibly a useful screening test if performed at the early stages of pregnancy. The impairment of NO metabolism in PE appears to play an important role in its pathogenesis; therefore, it is a potential therapeutic target.

## Figures and Tables

**Table 1:** Patient characteristics.

Variable	Control Group	PE Group	Statistical Significance
Age (years)			*p* = 0.0837
	27.74 + 4.72	29.71 + 6.10	
Primiparous			*p* = 0.8965
	23 (54.762%)	31 (53.448%)	
Miscarriages			*p* = 0.6084
	33 (78.571%)	43 (74.138%)	
BMI			*p* = 0.7741
	28.83 + 4.77	29.15 + 6.03	
Patient with Comorbidities			*p* = 0.0062
	5 (11.905%)	21 (36.207%)	
Gestational age (weeks)			*p* = 0.0518
	37.90 + 4.16	36.33 + 3.80	
Newborn weight (g)			*p* = 0.0071
	3117.38 + 873.43	2652.03 + 807.13	

**Table 2:** NOx concentrations.

		Control Group (µmol/L)	PE Group (µmol/L)
Mean		27.64	5.33
Standard deviation		18.06	7.01
Minimum		5.76	0
Maximum		100.42	33.87
*t*-test	*t*	7.6017	
	df	50.0085	
	*p*	<0.0001	

## Data Availability

Data and material are available upon at Maciej W. Socha request.
